# Identification of Common Regulators of Genes in Co-Expression Networks Affecting Muscle and Meat Properties

**DOI:** 10.1371/journal.pone.0123678

**Published:** 2015-04-14

**Authors:** Siriluck Ponsuksili, Puntita Siengdee, Yang Du, Nares Trakooljul, Eduard Murani, Manfred Schwerin, Klaus Wimmers

**Affiliations:** Institute for ‘Genome Biology’, Leibniz Institute for Farm Animal Biology (FBN), Wilhelm-Stahl-Allee 2, D-18196 Dummerstorf, Germany; University of Bologna, ITALY

## Abstract

Understanding the genetic contributions behind skeletal muscle composition and metabolism is of great interest in medicine and agriculture. Attempts to dissect these complex traits combine genome-wide genotyping, expression data analyses and network analyses. Weighted gene co-expression network analysis (WGCNA) groups genes into modules based on patterns of co-expression, which can be linked to phenotypes by correlation analysis of trait values and the module eigengenes, i.e. the first principal component of a given module. Network hub genes and regulators of the genes in the modules are likely to play an important role in the emergence of respective traits. In order to detect common regulators of genes in modules showing association with meat quality traits, we identified eQTL for each of these genes, including the highly connected hub genes. Additionally, the module eigengene values were used for association analyses in order to derive a joint eQTL for the respective module. Thereby major sites of orchestrated regulation of genes within trait-associated modules were detected as hotspots of eQTL of many genes of a module and of its eigengene. These sites harbor likely common regulators of genes in the modules. We exemplarily showed the consistent impact of candidate common regulators on the expression of members of respective modules by RNAi knockdown experiments. In fact, Cxcr7 was identified and validated as a regulator of genes in a module, which is involved in the function of defense response in muscle cells. Zfp36l2 was confirmed as a regulator of genes of a module related to cell death or apoptosis pathways. The integration of eQTL in module networks enabled to interpret the differentially-regulated genes from a systems perspective. By integrating genome-wide genomic and transcriptomic data, employing co-expression and eQTL analyses, the study revealed likely regulators that are involved in the fine-tuning and synchronization of genes with trait-associated expression.

## Introduction

Muscle is the major energy consumption and storage organ in animals. An imbalance in the supply and demand of nutrients, energy, and oxygen in muscle cells is evident in many diseases. Termination of nutrient and energy supply and anoxia in muscle cell also occurs *postmortem*. Physiological processes occurring to change from muscle to meat involve a gene expression pattern associated with both muscle structural and metabolic processes [1, 2]. Studies have begun to identify co-expression networks of genes involved in the functionality of muscles (contractile, metabolic, and structural properties) and their potential relationships to differences in muscle plasticity, size and shape, fibers as well as meat quality [3–5]. However, more work is needed to better understand the contribution of genetics to muscle-related phenotypes.

Systems genetics provide powerful tools to gain a systems-level perspective of genetic variation and reveal the molecular networks of complex traits. Indeed, this kind of approach has revealed some of the biology of muscle and meat characteristics and their related metabolic effects [6] as well as the role of genetic variation in cell function and disease [7, 8]. Systems genetics techniques include genome-wide association, expression quantitative trait locus (eQTL) discovery, causality modeling, and network analysis [6, 9, 10]. In particular, weighted gene co-expression network analysis (WGCNA) is useful for identifying biological networks [11]. WGCNA groups genes into modules based on patterns of co-expression that are often enriched for genes that share similar functions [12]. Genes within a module can be organized by connectivity. Highly connected genes are called “hubs”, and in a co-expression network hub genes are those that are most strongly correlated with the largest number of other module genes [13]. For example, in a recent study of chondrocyte differentiation, hub genes were demonstrated to be more likely genetically associated with Bone mineral density (BMD) than non-hub genes [14].

We previously constructed a muscle co-expression network using gene expression microarray profiles from porcine muscle and WGCNA [15]. In the current study, the modules of co-expression genes that are correlated with *post mortem* traits are further analyzed in order to obtain highly connected hub genes of these networks. Moreover, we identified eQTL for the genes assigned to modules that show association with meat quality traits (pH, conductivity, color, driploss). The objective of the present study is to identify common regulators of genes in the modules correlated with post mortem traits by using eQTL analysis of (1) each of the genes in the modules including the highly connected hub genes and of (2) the module eigengene values, i.e. a distinct value per module representing its first principle component. In particular eQTL for the highly connected hub genes are shown and regions are identified that comprise many eQTL of genes of trait-associated modules as well as of their eigengene values. These genomic sites thus represent hotspots of eQTL harboring likely common regulators of genes in the modules. We exemplarily demonstrate by RNAi knockdown experiments that the integration of genome-wide genomic and transcriptomic data enables detecting members of regulatory networks that are involved in tuning and synchronizing of trait-associated- and correlated-expression of genes.

## Material and Methods

### Animals, tissue collection, and phenotyping

Animal care and tissue collection procedures followed the guidelines of the German Law of Animal Protection, and the experimental protocol was approved by the Institutional Animal Care and Use Committee (IACUC) of the Leibniz Institute for Genome Biology (FBN). Samples used in the study were taken from animals that were slaughtered for human consumption at the Leibniz Institute for Genome Biology (FBN)’s slaughter house at an age of ~180 days. This study was based on genotyping records, expression profiles, and genome-wide association analyses done with performance-tested pigs from commercial herds of the crossbreed Pietrain × (German Large White × German Landrace) (n = 207; 110 castrates, 97 females) [15].

### Whole-genome scan and quality control

Illumina bead array technology was used to carry out all genotyping reactions in accordance with the manufacturer's protocol for the SNP Infinium HD assay (http://www.illumina.com). Genotyping was performed using the PorcineSNP60 BeadChip (Illumina Inc., San Diego, CA, USA). In brief, 200 ng of DNA were used for genome-wide amplification, fragmentation and hybridized to locus-specific 50mers on the surface of the microarray. 207 samples (muscle) were genotyped for 62,163 SNPs, and quality of the data was evaluated. Samples with call rates <95% were removed. Markers were excluded if they had low minor-allele frequency (MAF) <5%. Deviation from Hardy-Weinberg equilibrium was not considered because a three-way crossbreed pig population was used; deviation from Hardy-Weinberg equilibrium can be expected due to discordant allele frequencies in the parental breeds. The mean call rate of all samples was 99.8% ± 0.2.

Gene expression profiling of M. longissimus dorsi samples was done using the same animals. According to Affymetrix protocols, 500 ng of total RNA were reverse-transcribed into cDNA, transcribed into cRNA, and labelled using Affymetrix one cycle synthesis and labelling kit (Affymetrix, UK) to prepare antisense biotinylated RNA targets that were hybridized onto Affymetrix Porcine Snowball Array (GEO platform: GPL16569). The microarray data related to all samples were deposited in the Gene Expression Omnibus public repository (GEO accession number: GSE32112: GSM796045-GSM796251).

### eQTL detection

Log2-transformed expression levels in muscle of 207 individuals were used as traits for whole-genome association analysis with SNPs genotyped by a mixed-model analysis of variance using JMP Genomics (SAS Institute). Genotype, sire, and gender as well as *ryanodine receptor-1* (*RYR1*) genotype were used as fixed effects, carcass weight as a covariate, and slaughter day as random effects. Annotation and localization (Ensembl_Sscrofa_10.2) of SNP sites and probe sets enabled discrimination of *cis*- and *trans*-eQTL; in fact we defined an eQTL as ‘*cis’* if an associated SNP was located within an area less than 1 Mb from the probe set/gene. All other eQTL were considered as ‘*trans’*. Results are reported at thresholds of p<10^–5^ and p<10^–6^, which was used in many reports (http://www.genome.gov/gwastudies) [16–18]. A P-P-plot is shown in S1 Fig. A Bonferroni corrected level of significance of p = 0.05 is 0.9 x10^-10^, which is too stringent while taking into account the likely higher dependence among the expression traits (correlated expression) and among the SNP genotypes (linkage disequilibrium). In order to assess the number of false positives among the significant associations we calculated the FDR of genes in the WGCNA (weighted gene co-expression network analysis) modules that represent groups of co-expressed genes and are by default named by colors. A p<10^–5^ corresponds to FDR of 0.1 in the module named `dark-turquoise´ and 0.2 in the module named `orange´; a p<10^–6^ corresponds to FDR of 0.04 in module dark-turquoise and 0.1 in module orange.

### Weighted Gene Co-expression Network Analysis (WGCNA) and eQTL

A weighted gene co-expression network was constructed for 207 muscle biopsies using the blockwise modules function from the WGCNA package in R [11] (details are given in S1 Doc). Expression datasets comprising 11,191 probe sets out of 24,123 probe sets on the array were used for further analysis after quality control, filtering for present/absent calls and normalization. Residuals of gene expression, after correcting for the above-mentioned effects, were used for WGCNA. The blockwise modules function allows the entire dataset of 11,191 probe sets to be utilized in the construction of the weighted gene co-expression network (see Ponsuksili et al., 2013 for detailed methods) [15]. Modules were further merged based on the dissimilarity between their eigengenes, which were defined as the first principal component of each module. Module–trait associations were estimated using the correlation between the module eigengene and the phenotype, which allows easy identification of expression sets (modules) highly correlated to the phenotype [15]. Within each module, the intramodular connectivity of each gene was evaluated using two methods, defined as module membership (MM) and the soft connectivity (K). Module membership (MM) was defined as the correlation of expression profile (*x*_*i*_) and each module eigengene (ME),

$$MM_{}=cor(x_{i},ME),$$

The intramodular soft connectivity (K) was defined as,

$$K_{i}=\sum_{u\neq i}a_{iu},$$

which is the sum of all pairwise adjacencies of a gene to all other genes, *x*_*u*_, in the module. Genes within each module were then ranked using both the absolute value of module membership and the intramodular soft connectivity, which enables further identification of key players in the regulation network, defined as hub genes. When multiple transcripts exist for a single gene, the ranking of the gene was determined by the transcript with the highest module membership. The hub gene networks were drawn using Cytoscape [19].

**Fig 1:**
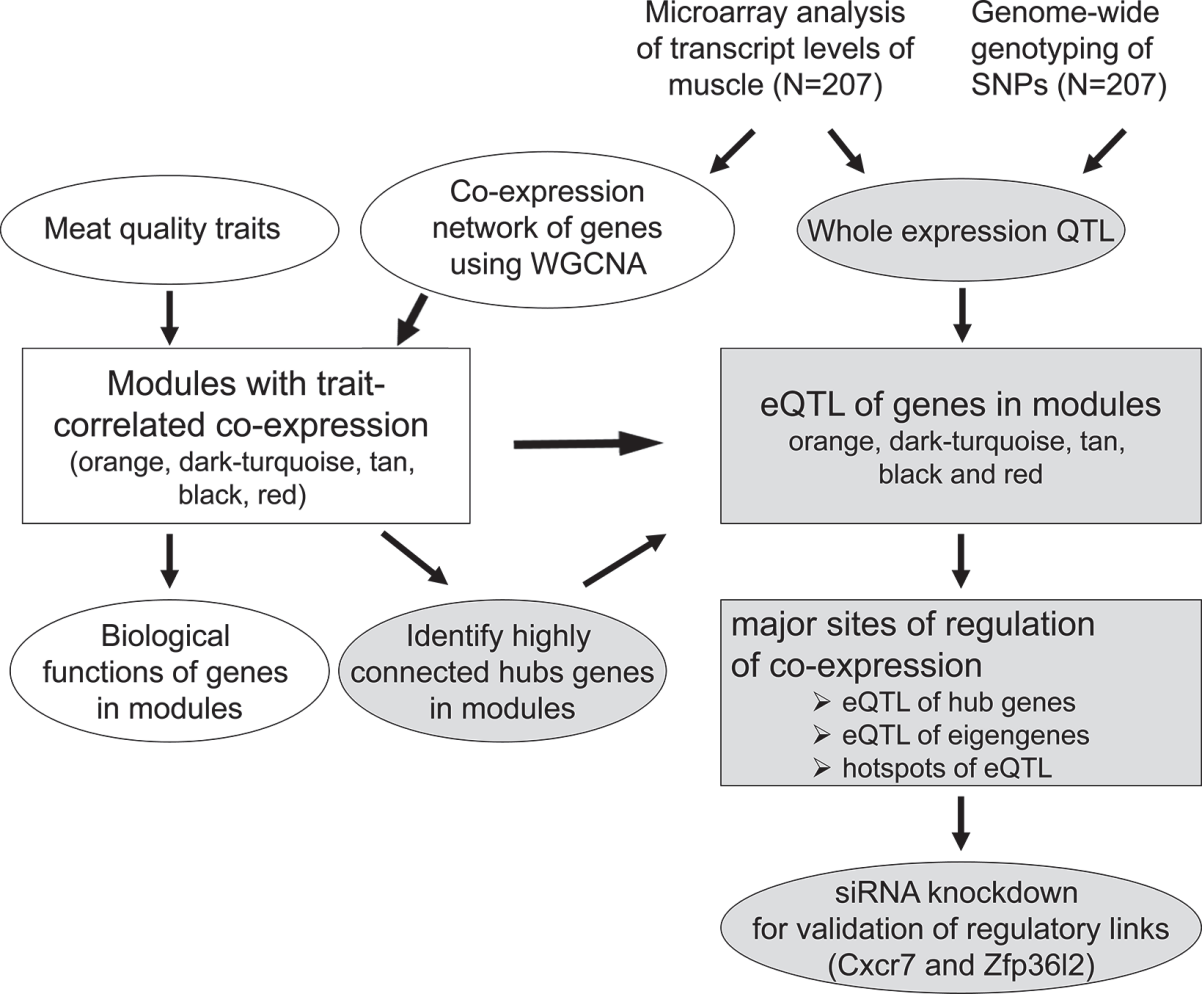
Outline of the systems biology approach to dissect the molecular networks of complex traits like meat quality. The transcript data were integrated with SNP genotype to map expression QTLs (eQTL) of all present transcripts as revealed by microarray analysis. Previously obtained co-expression networks which were correlated with meat quality were analyzed ([15]; white elements) in more depth to reveal the highly connected hub genes in the co-expression modules. Subsequently transcripts and corresponding eQTL of trait-associated modules were addressed. In order to reveal major sites of orchestrated regulation of these genes, eQTL for individual genes including the hub genes in the modules were detected and sites, where several eQTL of multiple genes existed, were considered as hotspots of eQTL harboring likely common regulators of genes in the modules. Moreover, the eigengene of each module was used for association analysis to identify common regulators of genes in module. Genes within the major sites of regulation were listed that were found to be correlated with the expression of genes in the respective module. Finally, the functional link of these potential regulators was exemplarily validated by siRNA knockdown.

To identify genetic loci responsible for the coordinated expression of genes in a module expressionQTL, eQTL, were detected. In addition to the individual expression levels of the genes in the modules, including the hub genes, also the eigengene of each module was used for association analysis and eQTL detection (Fig 1). Genotype, sire, and gender as well as *RYR1* genotype were used as fixed effects, carcass weight as a covariate, and slaughter day as random effects.

### Cell Culture and siRNA transfection

Mouse skeletal muscle C2C12 myoblasts passage no. 7–9 (ATCC) were grown and maintained in Dulbecco's modified Eagle's medium (DMEM, Invitrogen) containing 10% fetal bovine serum (PAA). At 80–90% confluences, myogenic differentiation was induced by switching to 2% horse serum-supplemented DMEM. Differentiation medium was changed every day during the course of myotube induction. All cultures were maintained under normal growth conditions (37°C and 5% CO_2_) and all media were supplemented with 1% penicillin/streptomycin (Biochrom).

Synthetic siRNAs were pre-designed by Qiagen. A total of 4 pre-designed siRNAs (Qiagen) per gene were first tested. The most effective siRNA for each gene was used (Mm_Zfp36l2_3 FlexiTube siRNA, Mm_Cmkor1_7 FlexiTube siRNA). The average values of negative non-silencing control siRNA (AllStars Negative Control siRNA, Qiagen), mock, and untreated were used as control. Transfection of siRNA was carried out using the HiPerFect transfection reagent (Qiagen) at 150 nM final concentration. Briefly, C2C12 cells were seeded in six-well plates at a density of 1.5 × 10^5^ cells/well with 2 mL growth medium. Twenty-four hours after seeding, the cells were changed to 2.3 mL of differentiation media. A transfection complex was prepared by diluting 150 nM siRNA in 100 μL DMEM without serum, and then adding 24 μL of HiPerFect transfection reagent to the diluted siRNA. The complexes were added drop-wise onto the cells, and the plates were then gently swirled to ensure uniform distribution of the transfection complexes. Forty-eight hours after siRNA transfection, cells were rinsed 2 times with PBS. The transfected cells were harvested for monitoring the effect of gene silencing. Four to six independent experiments were conducted.

We determined the level of knockdown of cDNA using quantitative PCR (qPCR) (Roche, Germany) and normalized data using *Hrrt1* and *Ppia* as an internal control. To use these two genes as a reference, geometric means of raw threshold cycle (CT) values of these two genes were used for further calculations. All statistical analyses were performed using two-tailed Student's t-tests.

## Results

### eQTL identification

Using 207 samples collected from performance-tested pigs of commercial herds of the crossbreed Pietrain × (German Large White × German Landrace), we conducted muscle expression profiling using GeneChip Porcine Genome Arrays (Affymetrix) and genome-wide genotyping using PorcineSNP60K BeadChip (Illumina). ExpressionQTL were identified for 11,199 probe sets and 47,524 single-nucleotide polymorphisms (SNPs) after quality control and normalization. Two types of eQTL were distinguished: those that mapped near (less than 1 Mb from) the gene encoding the transcript of interest (*in cis*) and those that map either more distant on the same or on another chromosomes (*in trans*). We annotated gene- and SNP-localizations using the most recent genomic map of *Sus scrofa* (Ensembl, Sscrofa_10.2). Two significance levels of eQTL were identified. Comparing results at the significance levels of p<10^–5^ (total number of eQTL 47,836) and p<10^–6^ (total number of eQTL 25,042) revealed that the number of eQTL *in cis* dropped by 27% (p<10^–5^: 11,751; p<10^–6^: 8,604), whereas the number of eQTL *in trans* dropped by 57% (26,012 to 11,180). The 7,628 SNPs explain 9 to 78% of the variation in transcript levels of *cis* eQTL and 12,401 SNPs explain 9 to 64% of the variation in transcript levels of *trans* eQTL at p<10^–5^ (Table 1).

**Table 1:** Numerical summary of the whole-genome association study of gene expression levels in muscle (eQTL).

	*p<10* ^*-6*^(probe sets, genes)	*p<10* ^*-5*^(probe sets, genes)
No. of samples	207	
Total probe sets	11199	
Total SNPs	47524	
No. of eQTL	25042 (2567, 2191)	47836 (6537, 5239)
No. of *cis*-eQTL*	8604 (1224, 1041)	11751 (1572, 1327)
No. of *trans*-eQTL	11180 (1552, 1371)	26012 (5024, 4119)

*Localization of SNP site and probe set sequences based on Sscrofa_10.2 genome sequence in 1-Mb windows; note that not all probe sets and SNPs are mapped to the genome; thus the number of eQTL assigned being either cis or trans is lower than the total number of eQTL

### eQTL integration with genes in modules

Estimates of eQTL of all quantified transcripts do not provide any link to organismal phenotypes of interest. In order to become focussed on genes and eQTL relevant to meat performance we sought to identify genetic loci that are associated with the variation of transcript abundance of co-expressed genes which showed trait-associated expression. In fact, among the co-expressed genes assigned to various so-called modules some were found to be highly correlated with *post mortem* traits [15]. These highly trait-correlated-genes belonged to co-expression networks modules named `black´, `red´, `tan´, `orange´, and `dark-turquoise´. A numeric summary of these eQTL is shown in Table 2. Modules black and red comprised 436 and 315 genes, respectively, that were mainly related to the GO terms `mitochondrion´ and `mitochondrial ribosome´. At p<10^–5^ there were 316 and 229 genes of the two modules that exhibited eQTL. Module tan, which was enriched for genes of the top functional annotation cluster `extracellular matrix´, had 83 genes with corresponding eQTL, with 12 genes having *cis* eQTL and 63 genes having *trans* eQTL at p<10^–5^. Module dark-turquoise was highly enriched for genes involved in `cell death or apoptosis´ and `carbohydrate metabolism´. There were 25 genes with eQTL, with 6 corresponding genes in *cis* and 20 in *trans* at p<10^–5^. Module orange comprised only 36 probe sets and was enriched for transcripts of the functional annotation cluster `defense response´. 19 out of 26 annotated genes had eQTL, 2 genes had *cis* and 19 genes had *trans* eQTL at p*<10* ^*–5*^.

**Table 2:** Number of eQTL for co-expressed genes of modules from muscle networks whose eigengene showed significant correlation with meat quality traits.

Module (# of probe sets, genes in	Total no. of eQTL (# of genes with eQTL)		No. of *cis*-eQTL (# of genes with eQTL)		No. of *trans*-eQTL (# of genes with eQTL)	
modules)	*p<10* ^*–6*^	*p<10* ^*–5*^	*p<10* ^*–6*^	*p<10* ^*–5*^	*p<10* ^*–6*^	*p<10* ^*–5*^
black (515, 436)	924 (131)	2113 (316)	321 (55)	460 (69)	436 (87)	1215 (241)
dark-turquoise (51, 31)	51(9)	174 (25)	26 (3)	49 (6)	21 (6)	99 (20)
orange (36, 26)	16 (9)	66 (19)	6 (2)	9 (2)	10 (7)	53 (19)
red (364, 315)	698 (94)	1328 (229)	255 (50)	340 (59)	307 (48)	705 (164)
tan (184, 154)	74 (19)	321 (83)	32 (9)	65 (12)	32 (13)	192 (63)

### Integration of genetics and highly connected hubs in modules

**Fig 2:**
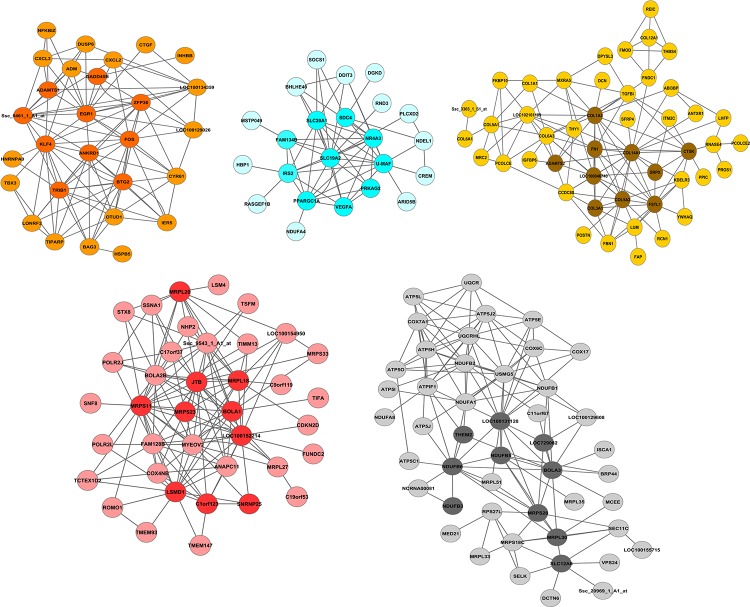
Networks of the top 150 correlated genes, including hub genes, in modules orange, dark-turquoise, tan, red, and black. Nodes of darker color within a module are the top 10 hub genes.

The correlation between each gene in a module and the module eigengene defined the eigengene-based connectivity. The top 150 correlated genes, including so-called hub genes with highest connectivity, of the modules of interest are shown in Fig 2. *EGR1*, *SLC19A2*, *COL14A1*, *C1orf123*, and *NDUFB6* were the highest connected genes in modules orange, dark-turquoise, tan, red, and black, respectively. These genes also showed the highest positive correlations of their expression levels and the eigengene (r = 0.86, p = 5.6x10^-61^; r = 0.90, p = 5.1x10^-75^; r = 0.93, p = 1.7x10^-92^; r = 0:90; p = 1.9x10^-74^; and r = 0.93, p = 6.9x10^-93^).

In order to be even more focussed on those genes that are involved in tuning and synchronizing trait-associated- and correlated-expression of the genes within the selected modules we considered the eQTL of the hub genes within these modules. Moreover, we estimated the genome-wide association of the eigengene of each of these modules. QTL of the hub genes and QTL of the eigengene potentially detect common regulators of the orchestrated and trait-correlated genes in the modules.

**Fig 3:**
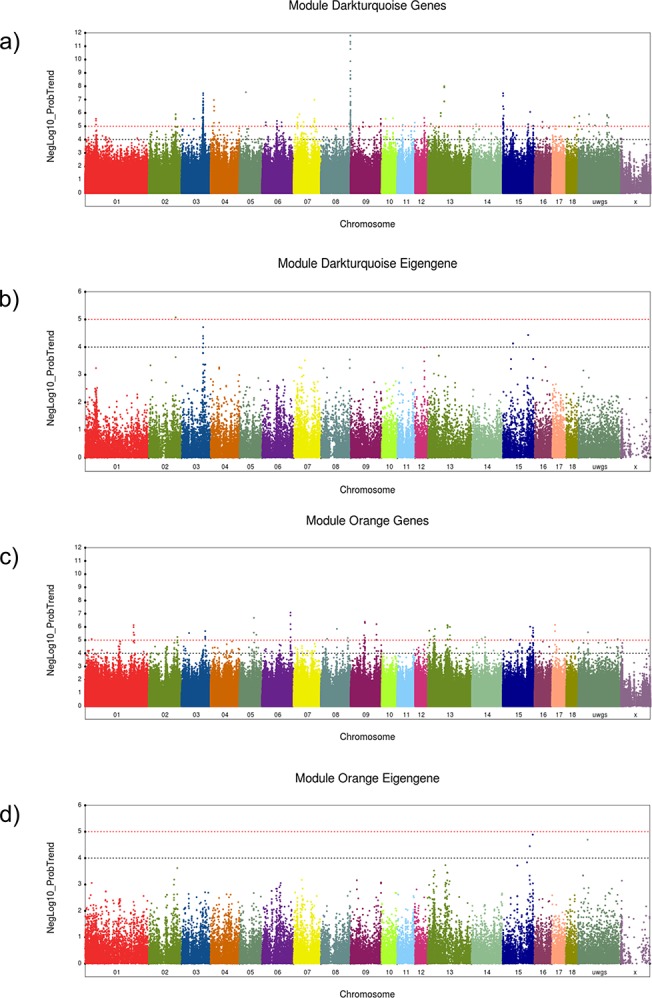
ExpressionQTL identified with the expression values of genes in the modules or the eigengene value of the respective module. Black and red dashed lines are a genome-wide significance threshold corresponding to negative log_10_ (NegLog10)>4 and >5, respectively. a-b, Manhattan plots of genome-wide association analyses of (a) transcript abundance of genes and (b) eigengene value for module dark-turquoise. c-d, Manhattan plots of genome-wide association analyses of (c) transcript abundance of genes and (d) eigengene value for module orange.

The genome-wide association of co-expressed genes in module dark-turquoise is shown in Fig 3A. The most significant eQTL was found for *RASGEF1B*, with *cis* effects at SSC8 position 145.9 Mb and a LOD score greater than 11. Other genes with *cis* or *trans* eQTL in this module were the most highly connected genes, for example, *SLC19A2*, *SLC20A1*, *FAM134B*, or *NR4A3* (Table 3). For association of the eigengene of module dark-turquoise and common genetic loci impacting the correlated-expression of module members, significance reached a level of p>10^–4^ (Fig 3B).

**Table 3:** Genes of module dark-turquoise that ranked top for intramodular connectivity according to module membership (MM) and soft connectivity (K) with *trans* or *cis* eQTL at *p*<10^–5^.

Gene symbol	SSC chromosome (Mb)	eQTL	MM	K
*SLC19A2*	4 (89.1)	*trans*	0.90	4.25
*NR4A3*	1 (269.8)	*trans*	0.87	4.50
*SLC20A1*	unknown	*trans*	0.86	4.39
*FAM134B*	16 (6.1)	*trans*	0.82	4.35
*PRKAG2*	18 (6.1)	*trans*	0.79	2.87
*BHLHE40*	13 (67.8)	*trans*	0.73	2.07
*NDEL1*	12 (56.1)	*trans*	0.72	1.44
*MSTP049*	18 (40.2)	*cis*	0.72	1.12
*PLCXD2*	13 (159.0)	*cis*	0.68	0.90
*CREM*	10 (63.5)	*trans*	0.66	1.39
*SDC4*	17 (53.2)	*trans*	0.66	0.95
*HBP1*	unknown	*trans*	0.66	0.84
*RND3*	15 (1.0)	*cis*, *trans*	0.65	1.37
*RASGEF1B*	8 (145.8)	*cis*,*trans*	0.64	1.03
*DGKD*	15 (147.9)	*trans*	0.63	0.79
*KIAA1217*	10 (56.1)	*cis*	0.62	0.94
*AGPAT6*	17 (12.1	*trans*	0.60	0.54
*FBXO32*	4 (16.6)	*cis*, *trans*	0.58	1.13
*NPC1*	6 (100.9)	*trans*	0.56	0.47

Association of SNPs with each single gene within the modules of co-expressed genes in module orange is shown in Fig 3C. Most of the genes with *cis* or *trans* eQTL at p<10^–5^ in this module were found to be the highly connected hub genes, like *EGR1*, *FOS*, or *ZFP36* (Table 4). QTL for the eigengene of module orange reached a significance level of *p* = 10^–4^ (Fig 3D).

**Table 4:** Genes of module orange that ranked top for intramodular connectivity according to module membership (MM) and soft connectivity (K) with *trans* or *cis* eQTL at p<10^–5^.

Gene symbol	SSC chromosome (Mb)	eQTL	MM	K
*EGR1*	2 (146.1)	*cis*, *trans*	0.86	3.41
*FOS*	7 (104.2)	*trans*	0.84	2.24
*ZFP36*	6 (43.8)	*trans*	0.84	1.98
*BTG2*	9 (70.3)	*cis*, *trans*	0.77	1.92
*KLF4*	1 (279.0)	*trans*	0.82	1.59
*OTUD1*	10 (57.2)	*trans*	0.74	1.53
*LOC100134359*	2 (66.2)	*trans*	0.73	1.46
*GADD45B*	2 (76.6)	*trans*	0.64	1.44
*LOC100129026*	16 (55.7)	*trans*	0.72	1.20
*ADAMTS1*	13 (200.1)	*trans*	0.72	1.10
*ADM*	2 (52.5)	*trans*	0.75	0.88
*TIPARP*	13 (104.6)	*trans*	0.65	0.74
*CXCL3*	8 (74.3)	*trans*	0.67	0.72
*CXCL2*	8 (74.3)	*trans*	0.63	0.65
*JUN*	6 (141.2)	*cis*, *trans*	0.65	0.45
*CTGF*	1 (35.1)	*trans*	0.64	0.45

For eigengenes of modules black red and tan no significant QTL were detected. Only the eigengene of modules orange and dark-turquoise revealed QTL at p<10^–4^. Therefore further studies focused on these two modules that were highly correlated with *post mortem* phenotypes.

### A regulator of genes in module dark-turquoise

The eigengene of each module was used for association analysis, in order to identify genetic loci accountable for the coordinated expression of genes within modules. The most prominent associated SNP region of the eigengene in module dark-turquoise was found on chromosome 3 (Fig 3B). This position on chromosome 3 also showed an accumulation of eQTL for genes of the module. QTL for the eigengene and for many of the highly connected genes of the module confirmed the distal region of SSC3 as a major site of regulation of co-expression of module dark-turquoise genes (Fig 3A and 3B). In fact, this eQTL region of the eigengene of module dark-turquoise covered 4 SNPs (ASGA0015591, ALGA0020437, ASGA0097215, MARC0053416) located at chromosome 3 between 106.8 to 107.7 Mb. A total of 6 genes (*SLC19A2*, *PPARGC1A*, *PLCXD2*, *NR4A3*, *IRS2*, and *FAM134B*) corresponding to 37 eQTL were found to be associated with major these 4 SNPs. This region represents a kind of hotspot of *trans*-eQTL of genes of module dark-turquoise.

We further focused on genes located in this region of SSC3 (using Sscrofa_10.2 map) that may be regulators of the 6 genes. We hypothesized that regulatory genes in this region that affect genes in module dark-turquoise should correlate with expression levels of these genes in module dark-turquoise. Probe sets of the Affymetrix chip detected 25 transcripts of genes located to SSC3 (103–108 Mb). We calculated the correlation of these 25 transcripts with the eigengene of module dark-turquoise. In total, we found 8 genes in this region with r > |0.20| at p < 0.001 (Table 5). The highest correlation of genes in this region with the eigengene of module dark-turquoise was seen for *ZFP36L2*, with r = 0.63 (p = 1.6 x10^-24^).

**Table 5:** Genes located at 103–109 Mb of SSC3 and correlated with the eigengene of module dark-turquoise.

Gene symbol	Position (Mb)	r	p-value	eQTL	Gene name
*THADA*	103.3	0.25	2.9x10^-4^	*trans*	Thyroid adenoma-associated protein homolog
*ZFP36L2*	103.3	0.63	1.6x10^-24^	*trans*	zinc finger protein 36, C3H type-like 2
*EML4*	104.1	0.31	3.9x10^-6^	*trans*	Echinoderm microtubule associated protein like 4
*CDKL4*	107.2	0.27	6.1x10^-5^	*trans*	cyclin-dependent kinase-like 4-like
*SOS1*	107.5	0.22	1.3x10^-3^	*cis*	son of sevenless homolog 1 (Drosophila)
*LOC375196 (Ssc_26576_1_A1_at)*	107.5	-0.28	4.2x10^-5^	*cis*	non-coding RNA
*SFRS7*	107.8	0.21	1.8x10^-3^	*trans*	serine/arginine-rich splicing factor 7-like
*CDC42EP3*	108.9	-0.26	1.9x10^-4^	*cis*	cdc42 effector protein 3-like

**Fig 4:**
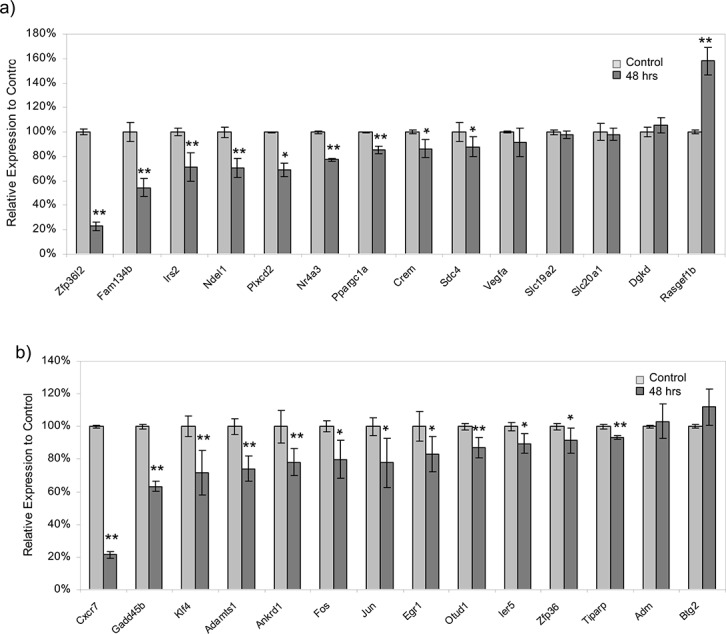
Knockdown of regulator genes by RNA interference reveals regulation within modules. siRNAs were designed to target regulator genes in modules dark-turquoise and orange, and transfected into murine C2C12 muscle cells *in vitro*. Relative mRNA expression was measured by qPCR 48 hours after transfection. Expression was normalized to *Hrrt1* and *Ppia* internal controls. a) *Zfp36l2* as a partial regulator of genes in module dark-turquoise. Expression of *Zfp36l2* was significantly reduced relative to its expression in control cells at 48 hours post-transfection of siRNA. Expression of other genes (*Fam134b*, *Irs2*, *Ndel1*, *Nr4a3*, *Ppargc1a*, *Crem*, *Sdc4*) in module dark-turquoise was significantly reduced; these genes are functionally enriched in apoptosis and cell death. b) *Cxcr7* as a regulator of genes in module orange. siRNA targeting significantly reduced the levels of *Cxcr7* relative to control at 48 hours post-transfection. Expression of most top hub genes in module orange, like *Egr1*, *Zfp36*, *Fos*, *Klf4*, *Ankrd1*, *Otud1*, *Adamts1*, *Gadd45b*, *Ier5*, *Tiparp*, and *Jun*, was also reduced. The data represent mean±SEM (n = 4–6 independent experiments). * indicated significant level at p<0.05 and ** indicated significant level at p<0.01.

To validate the association of this gene with the expression of genes of the module dark-turquoise, RNAi was used to knockdown *Zfp36l2* expression *in vitro* in the C2C12 murine muscle cell line. Subsequently, relative expression of *Zfp36l2* as well as 13 other genes in module dark-turquoise was measured using qPCR. siRNA targeting *Zfp36l2* effectively inhibited its expression to 23% (p = 1.3x10^-5^) relative to control cells. Further, *Zfp36l2* inhibition resulted in relative expression changes of 9 out of 13 genes of the module (p<0.05) (Fig 4A). Of these 9 genes, 7 (*Fam134b*, *Irs2*, *Ndel1*, *Nr4a3*, *Ppargc1a*, *Crem*, *Sdc4*) were related to the biofunctions apoptosis and cell death.

### A regulator of genes in module orange

Three SNP regions (139.7 Mb (DIAS0000052), 149.2 Mb (ASGA0071618), and 150 Mb (ASGA0094233) on SSC15 circumscribe hotspots of eQTL for members of module orange and its eigengene and were found to harbor likely regulators of genes of module orange. We calculated the correlation of genes in this region with the eigengene of module orange (Table 6). No transcript located at region 139 Mb was found to have significant correlation with the transcript abundance of genes in module orange. However, the expression level of *CXCR7*, located at 150.8 Mb of SSC15, was highly correlated with the eigengene of module orange (r = 0.49 with p<1.2x10^-13^). Moreover, *ARL4C*, located on SSC15 at position 149.1 Mb, was also significantly correlated with genes in module orange (r = 0.20 with p<4.5x10^-3^).

**Table 6:** Genes located at 134 Mb and 149–150 Mb of SSC15 and correlated with the eigengene of module orange.

Gene symbol	Position (Mb)	r	p-value	eQTL	Gene name
*ZFAND2B*	134.3	-0.30	1.5x10^-5^	*trans*	AN1-type zinc finger protein 2B
*ABCB6*	134.3	-0.28	4.8x10^-5^	*cis*, *trans*	ATP-binding cassette, sub-family B, member 6
*DNPEP*	134.4	-0.26	1.6x10^-4^	no	aspartyl aminopeptidase
*ARL4C*	149.1	0.20	4.5x10^-3^	no	ADP-ribosylation factor-like 4C
*CXCR7*	150.8	0.49	1.2x10^-13^	*trans*	chemokine (C-X-C motif) receptor 7

We used RNAi to knockdown *Cxcr7* expression in C2C12 muscle cells *in vitro* and tested its expression and expression of other genes in module orange with qPCR. siRNA targeting of *Cxcr7* reduced its expression to 21% (p = 2.3x10^-6^) of the level seen in control cells. Further, 11 out of 13 other genes in the module differed significantly relative to control cells (p<0.05) (Fig 4B).

## Discussion

Attempts to dissect complex quantitative traits, in order to elucidate their genetic foundation, aim at collecting multiple evidence for the role of pathways and genes. Therefore, genome-wide genotyping, expression data analyses and network analyses are combined. Accordingly, we have previously identified QTL regions for muscle and meat traits by GWAS. SNPs were addressed that were associated with these muscle properties and with the expression of genes that show trait-correlated transcript abundance themselves (cis eQTL) and map within the QTL regions [20]. Likewise, we have previously deduced 22 co-expression networks for muscle, each of which contained genes that shared similar expression patterns and were enriched for functionally similar genes [15]. Co-expression modules named dark-turquoise, orange, red, black, and tan were strongly associated with traits. Highly connected network hub genes of the modules and common regulators of genes in the modules are likely to play an important role in the emergence of respective traits and are central to the network’s architecture [21, 22]. Therefore, here we identified eQTL for the genes, which were assigned to modules showing association with meat quality traits, including the highly connected hub genes. Moreover, the module eigengene values were used for association analyses in order to derive a joint eQTL for the respective module. Thereby major sites of orchestrated regulation of genes within trait-associated modules were detected as hotspots of eQTL of many genes of a module and of its eigengene.

Recent studies have demonstrated that co-expression modules can be conserved across species [8]; therefore, it is of significant interest to know if the relationships of genes in porcine muscle occur similarly in a mouse muscle cell line (C2C12). Our study confirms that co-expression of genes in modules is conserved across species between porcine muscle and mouse muscle. We identified genetic loci responsible for co-expression. The eigengenes of modules were used for identification of genetic regulation hot spots that specifically affect genes of the co-expression networks. Eigengenes of modules dark-turquoise and orange reached the significance level of *p*<10^–4^. We therefore focused on those two modules, which were predicted to play biologically significant roles in muscle cells and finally muscle pathology or meat quality. We discovered that *CXCR7* was the key driver of module orange gene expression, which is involved in the function of defense response in muscle cell. *ZFP36L2* was identified as a partial regulator in module dark-turquoise, which plays a significant role in cell death or apoptosis.

### Highly connected hub genes are central to the network’s architecture

Recently, a number of studies have shown that highly connected hub genes tend to play significant roles in module organization and might be expected to play the most influential regulatory roles [8]. Many hub genes in this study, like *BTG2*, *EGR1*, *ANKRD1*, and *FOS*, were previously confirmed as transcriptional regulators in myogenesis [23]. *BTG2* expression was reported to play a key role in skeletal muscle growth and fat traits in pig [24, 25]. Knockdown of *Btg2* using lentiviral–based shRNA and siRNA severely impaired myotube formation through cell-cycle arrest [23, 26]. Early growth response transcription factor (*EGR1*) is an immediate early response gene with a zinc finger transcription factor that functions to drive many biological processes such as differentiation, proliferation, inflammatory response, and muscle regeneration during skeletal muscle wound healing [27, 28]. This confirms our finding that *EGR1* was the most connected hub gene with *cis* and *trans* mQTL effects in module orange. The second-most highly connected gene involved in myogenesis was *FOS* [29, 30]. The cellular *FOS* (*c-Fos*) is a cellular proto-oncogene belonging to the immediate early gene family of transcription factors. The QTL for skeletal muscle fiber and metabolism traits have been mapped to the marker interval around *FOS* and may underlie phenotypic variation in skeletal muscle fiber and metabolism traits in the pig [31]. Another gene in module orange, *GADD45B*, encodes a ubiquitously expressed protein that is often induced by DNA damage and other stress signals associated with growth arrest and apoptosis [32]. *GADD45B* plays an essential role in the apoptotic death of cardiomyocytes during ischaemic/hypoxic injury [33]. Most of the genes in this module belong to a group of early response genes that are stimulated by many environmental signals including growth and differentiation factors, hormones, neurotransmitters and physical stresses, like injury, heat, and radiation. These genes, then, may function as a convergence point for many signalling cascades, as suggested by their high connectivity.

Energy metabolism in muscle cell plays a significant role to final meat quality. The shifting from aerobic to anaerobic metabolism results in a pH decline *post mortem* and thereby influences meat qualities like drip loss and color [34]. Many hub genes in modules red and black have a significant function in energy metabolism in muscle. Muscle structure also plays a significant role in *post mortem* traits [15]. The genes in module tan were enriched in the GO category of “extracellular matrix” and collagens are its major constituents. Eight out 20 top hub genes in this module were collagen genes. The collagen network is also required in the muscle, not only for structural support but also for controlling cellular processes. *COL14A1* was identified as the most connected gene in this module. There was evidence that type XIV collagen mRNA increased in the muscle connective tissues after denervation and around the regenerating muscle fibers [35]. Recently, *Col14a1(-/-)* mice were found to exhibit significant perturbations in mRNA levels of many other collagen types and this study highlights the importance of the collagen network for myocardial cell survival, and function of the working myocardium after birth [36].

Some of the hub genes in module dark-turquoise (*SLC19A2*, *PRKAG2*, *IRS2*, *NR4A3*, *PPARGC1A* and *VEGFA*) involved in carbohydrate metabolism. Other genes in module were related to the apoptosis and cell death *(FAM134B*, *IRS2*, *NDEL1*, *NR4A3*, *PPARGC1A*, *CREM* and *SDC4*).

*SLC19A2* encodes the thiamin transporter protein. Thiamin plays a role in reducing cellular oxidative stress via its role in bridging the energy-producing glycolytic and the pentose phosphate metabolic pathway that is critical for creating chemical reducing power in cells [37–39]. Therefore, low levels of thiamine in cells lead to impairment in energy metabolism and to oxidative stress. Impaired links of glucose metabolites to the pentose pathway, result in depletion of reducing agents and accumulation of glycolysis end-products, which have deleterious effects. More thiamine is found in pork compared to meat of other species [40]. Thiamine is also a well-known meat flavor precursor [41]. Meat color and pH at 24 hours postmortem were correlated most with the gene in module dark-turquoise [15]. This implies that thiamin is not only essential for muscle cellular function but also for the meat quality post mortem.

Prkag2 encodes the AMP-activated protein kinase (AMPK) γ2 regulatory subunit. Histological studies of myocardial tissue and transgenic mouse models expressing mutant *Prkag2* confirmed glycogen storage as the pathologic basis for this cardiac syndrome [42]. Skeletal muscle AMP-activated protein kinase (AMPK) γ3 regulatory subunit, produces the dominant Rendement Napole (RN) phenotype identified in Hampshire pigs, which is caused by a single missense mutation (R225Q) in the AMPK γ3-subunit [43]. RN pigs have a 70% increase in glycogen content in skeletal muscle, whereas liver and heart glycogen contents remain unchanged [44]. In transgenic mice with an R225Q mutation in the γ3 subunit of AMPK, skeletal muscle showed increased expression of glycogen synthesis genes [45]. The glucose uptake in skeletal muscle has a large effects on meat characteristics [46, 47]. Insulin signaling plays a pivotal role in the regulation of glucose uptake by skeletal muscle [48]. Recently, IRS2 was shown to be regulated by miR-135a and that this interactions regulate skeletal muscle insulin signaling [49].

### Module regulator genes

Recent studies have begun identifying module quantitative trait loci and module regulator genes [8, 50]. Here, we identified *ZFP36L2* and *CXCR7* as genes with eQTL regulation in modules dark-turquoise and orange eigengene. This represents one of the first successful attempts at identifying the molecular basis of an eQTL.

ZFP36L2 is a member of the tristetraprolin (TTP, or ZFP36) family of CCCH-type tandem zinc finger proteins. These proteins can bind to transcripts containing AU-rich elements (AREs), resulting in deadenylation and destabilization of bound transcripts [51]. The active control of mRNA degradation has emerged as a key regulatory mechanism required for proper gene expression in the immune system [52]. Several RNA-binding proteins act through AU-rich elements to post-transcriptionally regulate the biosynthesis of proteins involved in inflammation [53]. The RNA-binding protein ZFP36L2 is a transcriptional target of the glucocorticoid receptor in burst-forming unit-erythroid (BFU-E) progenitors and is required for BFU-E self-renewal [54]. Targeted disruption of *ZFP36L2* results in defective hematopoiesis [53]. Many genes in module dark-turquoise like *SLC19A2*, *NR4A3*, *FAM134B*, *NDEL1*, *IRS2* contain AU-rich elements which may be targets of *ZFP36L2*. These genes were also found in AU-rich element-containing mRNA database of human [55, 56]. Module dark-turquoise co-expression genes are highly enriched for cell death or apoptosis and carbohydrate metabolism. Some of these genes belong to both categories. *ZFP36L2* was identified as a regulator of genes in module dark-turquoise. In fact, *ZFP36L2* regulated genes specifically having cell death or apoptosis functions rather than genes related to carbohydrate metabolism. The RNA-binding protein ZFP36L2 may regulate genes by acting through AU-rich elements of genes in this category.

*CXCR7* was identified as a regulator of genes in module orange in this study. It is a G-protein-coupled receptor with conserved motifs characteristic of chemokine receptors and it actively signals through the ß-arrestin pathway. CXCR7 is expressed in developing and adult mouse limb muscles and can control muscle growth during development and regeneration by abrogating CXCR4 signaling [57]. *CXCR7* is involved in cardiac valve remodeling possibly by regulating bone morphogenetic protein (BMP) signaling [58]. In C2C12 muscle cells, inhibition of *CXCR4* and *CXCR7* signaling, either by drugs or RNA interference, blocks myogenic differentiation [59]. The large number of chemokines and chemokine receptors directly expressed by muscle cells suggests that these proteins, and their immunological functions, might have a greater role in myogenesis or muscle regeneration than previously appreciated [60–64]. As shown in our previous study, module orange (26 annotated genes) was enriched for transcripts of the functional annotation clusters “response to wounding”, “defense response”, and “inflammatory response” and was highly significantly correlated with *post mortem* traits like pH and drip loss (pH24MLD r = 0.32, p = 3.7×10^−6^, DL r = -0.31, p = 5.8×10^−6^). The known role of *CXCR7* in muscle and the biological functions of genes in this module promote *CXCR7* as a major regulator of genes in module orange. Further work is needed to elucidate the mechanisms whereby *CXCR7* regulates transcripts in the module.

In this study, we integrated the detection of trait-related co-expression modules with genetic genomic analyses to detect eQTL for the genes in these modules, in particular for highly connected hub genes that are central to the network’s architecture as well as for the eigengenes. These data improve our understanding of the gene networks important for muscle function and demonstrate the ability of systems genetics to unravel gene networks involved in complex cellular processes.

## Supporting Information

S1 Doc Definitions and working steps. (DOCX)

S1 Fig P–P plot (probability–probability plot) of genome-wide association analyses of (a) transcript abundance of genes and (b) eigengene value for module dark-turquoise. c-d, P-value of quantile plotter of genome-wide association analyses of (c) transcript abundance of genes and (d) eigengene value for module orange.
(TIF)

S2 Fig Hierarchical clustering of modules eigengenes. (TIF)
