# Supplementary material for: Identification of Common Regulators of Genes in Co-Expression Networks Affecting Muscle and Meat Properties
Source: PLoS One. 2015 Apr 14;10(4):e0123678. doi: 10.1371/journal.pone.0123678 (PMC4397042; doi:10.1371/journal.pone.0123678)
Supplement: S1 Doc — (DOCX) [file pone.0123678.s001.docx]

Supplemental file: “**Definitions and working steps”**

The WGCNA (weighted gene coexpression network analysis) (Langfelder and Horvarth, 2008) procedure calculates an unsigned expression Pearson's correlation matrix for all genes, transforms the correlation matrix by raising all values to a power ß, calculates a topological overlap matrix from the transformed correlation matrix, converts the topological overlap matrix into a dissimilarity matrix, creates a hierarchical cluster tree based on the dissimilarity matrix, and identifies gene coexpression modules from the hierarchical cluster tree using a dynamic tree cut procedure. Accordingly, modules are coexpressed genes which were classified in the same groups.

A hierarchical cluster of the obtained modules is shown in S3. Eigengenes were defined as the first principal component of each module. Module–trait associations were estimated using the correlation between the module eigengene and the phenotype, which allows easy identification of expression sets (module) highly correlated to the phenotype.

Module membership (MM) was defined as the correlation of the expression profile and each module eigengene.

In brief, the steps of the analysis from the construction of weighted genes coexpression networks to the eigengene trait correlation matrix are as follows:

- A Pearson correlation matrix of each pairwise correlation between gene expression levels was calculated.
- The matrix was transformed using a power function resulting in a weighted network.
- Using this connection, genes were clustered in distinct groups (modules).
- The module eigengene values were calculated as the first principal component of the gene expression profiles in each module
- Modules eigengene values were used to estimate correlations with the traits of interest

Ref.:

Langfelder P, Horvath S (2008) WGCNA: an R package for weighted correlation network analysis. BMC Bioinformatics: 559
