# Supplementary figures and images for: Identification of Common Regulators of Genes in Co-Expression Networks Affecting Muscle and Meat Properties
Source: PLoS One. 2015 Apr 14;10(4):e0123678. doi: 10.1371/journal.pone.0123678 (PMC4397042; doi:10.1371/journal.pone.0123678)

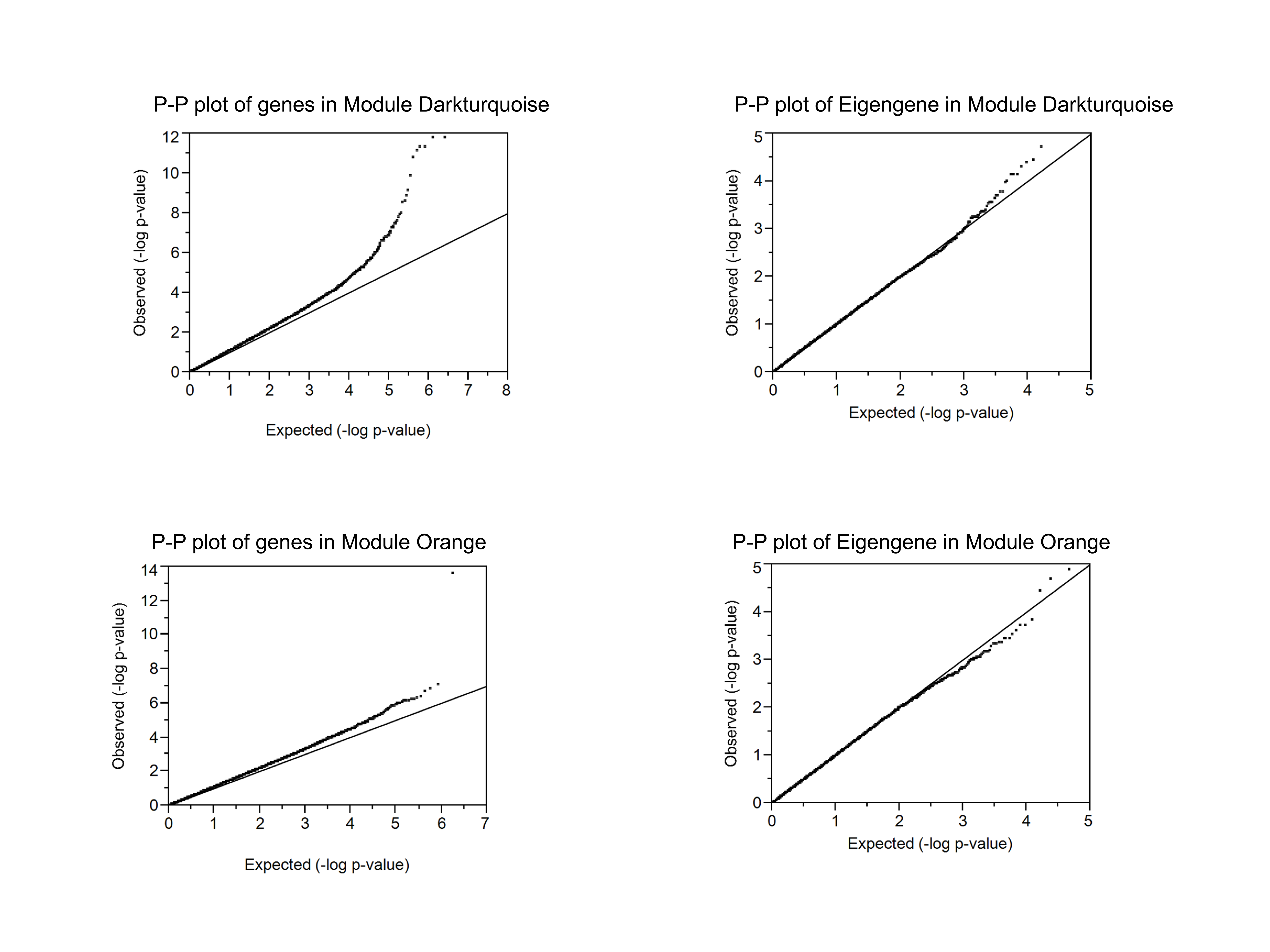

Supplement: S1 Fig — c-d, P-value of quantile plotter of genome-wide association analyses of (c) transcript abundance of genes and (d) eigengene value for module orange. (TIF) [file pone.0123678.s002.tif]

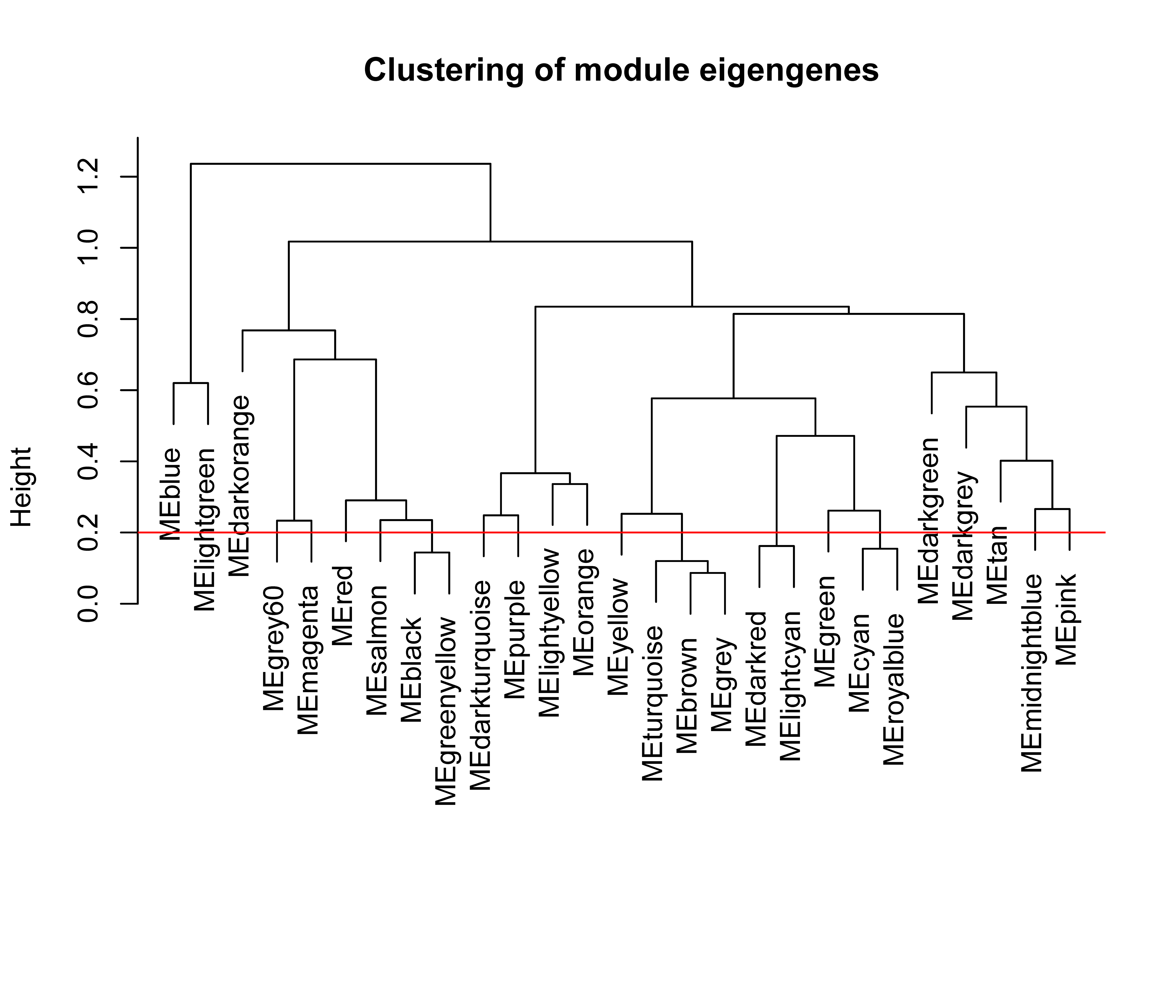

Supplement: S2 Fig — (TIF) [file pone.0123678.s003.tif]
